# *Myrica rubra* Extracts Protect the Liver from CCl_4_-Induced Damage

**DOI:** 10.1093/ecam/nep196

**Published:** 2011-02-14

**Authors:** Lizhi Xu, Jing Gao, Yucai Wang, Wen Yu, Xiaoning Zhao, Xiaohe Yang, Zengtao Zhong, Zhong-Ming Qian

**Affiliations:** ^1^Jiangsu Key Laboratory of Molecular Medicine, School of Medicine, Nanjing University, Nanjing, China; ^2^School of Pharmacy, Jiangsu University, 301 XUE Fu Road Zhenjiang, PRC 212013, Zhenjiang, China; ^3^Department of Experimental Radiation Oncology, The University of Texas M. D. Anderson Cancer Center, Houston, TX, USA; ^4^Life Science College, Nanjing Agricultural University, China; ^5^Laboratory of Iron Metabolism, Department of Applied Biology and Chemical Technology and Shenzhen Key Laboratory of Chinese Medicine and Molecular Pharmacology, The Hong Kong Polytechnic University, Kowloon, Hong Kong

## Abstract

The relationship between the expression of mitochondrial voltage-dependent anion channels (VDACs) and the protective effects of *Myrica rubra* Sieb. Et Zucc fruit extract (MCE) against carbon tetrachloride (CCl_4_)-induced liver damage was investigated. Pretreatment with 50 mg kg^−1^, 150 mg kg^−1^ or 450 mg kg^−1^ MCE significantly blocked the CCl_4_-induced increase in both serum aspartate aminotransferase (sAST) and serum alanine aminotransferase (sALT) levels in mice (*P* < .05 or .01 versus CCl_4_ group). Ultrastructural observations of decreased nuclear condensation, ameliorated mitochondrial fragmentation of the cristae and less lipid deposition by an electron microscope confirmed the hepatoprotection. The mitochondrial membrane potential dropped from −191.94 ± 8.84 mV to −132.06 ± 12.26 mV (*P* < .01) after the mice had been treated with CCl_4_. MCE attenuated CCl_4_-induced mitochondrial membrane potential dissipation in a dose-dependent manner. At a dose of 150 or 450 mg kg^−1^ of MCE, the mitochondrial membrane potentials were restored (*P* < .05). Pretreatment with MCE also prevented the elevation of intra-mitochondrial free calcium as observed in the liver of the CCl_4_-insulted mice (*P* < .01 versus CCl_4_ group). In addition, MCE treatment (50–450 mg kg^−1^) significantly increased both transcription and translation of VDAC inhibited by CCl_4_. The above data suggest that MCE mitigates the damage to liver mitochondria induced by CCl_4_, possibly through the regulation of mitochondrial VDAC, one of the most important proteins in the mitochondrial outer membrane.

## 1. Introduction

*Myrica rubra* Sieb. Et Zucc. is a myricaceae plant broadly distributed in eastern Asia. The leaves, bark and fruits of the tree have been used as astringent, antidote, and antidiarrhetic in traditional Chinese medicine [1, 2]. Several flavonoids, tannins [3], triterpenes [4] and diarylheptanoids [1, 5] have been isolated from the bark of *M. rubra* previously. In the pharmacological studies of this natural medicine, it has been reported that the extract of *M. rubra* bark exerts hepatoprotection [6, 7], inhibits melanin biosynthesis activities [8] and could prevent carcinogenesis [9]. But the hepatoprotective effects of *M. rubra* fruits are not well investigated.

Liver injuries induced by carbon tetrachloride (CCl_4_) are the best-characterized system of xenobiotic-induced hepatotoxicity and commonly used models for the screening of antihepatotoxic and/or hepatoprotective activities of drugs [10]. CCl_4_ causes mitochondrial stress, which activates signaling cascades involving the activation of caspases, resulting in apoptosis or necrosis. It is known recently that mitochondria in cells not only provide ATP by oxidative phosphorylation but also play many other roles, such as modulation of intracellular Ca^2+^ homeostasis, pH control and induction of apoptotic and excitotoxic cell death [11, 12]. There is accumulated evidence that mitochondrial permeability transition pore (PTP) plays a key role in modulating apoptotic and excitotoxic cell death. As a part of the outer membrane of mitochondria, the voltage-dependent anion channel (VDAC) is an important protein that regulates basic mitochondrial functions as well as the initiation of apoptosis via the release of intermembrane space proteins [13]. Our previous studies showed that both transcription and translation of liver VDAC changed significantly and both accompanied the mitochondrial damage in liver-damaged mice, which could be prevented by natural products [14].

In the present study, we evaluated the hepatoprotective effect of *M. rubra* Sieb. Et Zucc. extract (MCE) against liver injury induced by CCl_4_, addressing the possible action of MCE on liver mitochondrial and VDAC expression in order to search for the mechanism underlying its hepatoprotective activity.

## 2. Methods

### 2.1. Plant Material

**Figure 1:**
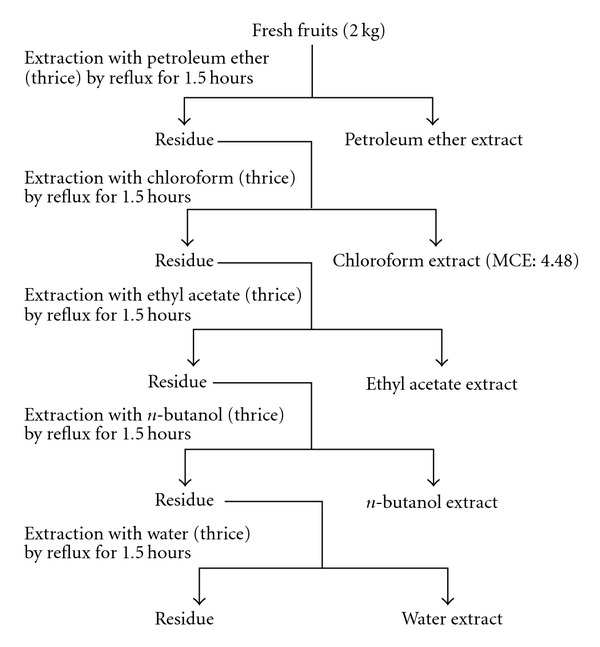
Reflux extraction of fresh fruit of *M. rubra* Sieb. Et Zucc. by increasing order of solvent polarity.

The chloroform extract of the fresh fruit of *M. rubra*—cultivated in Zhe Jiang province, China, and identified by Dr Yunzhi Zhu, Life Science College, Nanjing Agricutural University—was used in this study. Briefly, 2 kg of the fresh fruit was used for the chloroform extraction. It was prepared by adding solvents, namely petroleum ether, chloroform, ethyl acetate, *n*-butanol and water (Figure 1), in an increasing order of solvent polarity. After filtering through folded filter paper (Whatman No. 1), the supernatant in different solvents was recovered and this process was repeated thrice for each solvent. Then the respective solvents from the supernatant were evaporated in a vacuum rotary evaporator to obtain a crude extract. The dried form of the chloroform extract (MCE, 4.486 g) dissolved in 0.9% NaCl was used orally for checking of the hepatoprotective activities in mice.

### 2.2. Chemicals

Rhodamine123 (Rh123), succinate, rotenone and anti-VDAC antibody were purchased from Sigma (St Louis, MO, USA). RNAiso reagent, dNTP and Taq polymerase were from TaKaRa Biotechnology Co., Ltd. M-MLV reverse transcriptase was from Invitrogene. RNase inhibitor and Oligo(dT)_15_ were from Promega. All other chemicals were of high purity from commercial sources.

### 2.3. Animals

Male ICR mice (Experiment Animal Center of Yangzhou University, Yangzhou, China, Certificate No. SCXK 2003-0002), each weighing 18–22 g, were used. All animals were fed a standard diet *ad libitum* and housed at a temperature of 20–25°C under 12-h light-dark cycles throughout the experiment. All mice were acclimatized to the experimental conditions for 2 days before the start of the experiment, and they were randomly assigned to any one of the five groups. The Animal Ethics Committee of the Nanjing University approved the use of animals for this study.

### 2.4. CCl_4_-Induced Hepatotoxicity in Mice

Mice were allocated to any one of the five groups, each with eight animals. All mice except the normal ones received 0.30% of CCl_4_ (in olive oil, 10 mL kg^−1^ intraperitoneally, i.p.). The normal and CCl_4_ groups respectively received olive oil (10 mL kg^−1^ i.p.) and CCl_4_ following 5 days of oral treatment with saline [15, 16]. The drug groups received CCl_4_ following 5 days of oral treatment with MCE_L_, MCE_M_, and MCE_H_, representing 50, 150, or 450 mg kg^−1^ MCE [17, 18]. Mice were humanely killed 24 h after the administration of CCl_4_ and their blood was collected. Serum was separated by centrifugation, and serum aspartate aminotransferase (sAST) and serum alanine aminotransferase (sALT) activities were estimated spectrophotometrically. After blood draining, the liver sections were taken and fixed in 4% neutral-buffered formalin or in 4% glutaraldehyde containing 3% paraformaldehyde, and prepared for examination under a photomicroscope or electron microscope (JEM-1200EX) following standard techniques. The remaining liver lobes intended for mRNA and protein analyses were frozen immediately and stored in liquid nitrogen before extraction.

### 2.5. Isolation of Liver Mitochondria

Mitochondria were prepared from mouse livers according to the method of Aprille [19]. In brief, mouse livers were excised and homogenized in isolation buffer containing 225 mM d-mannitol, 75 mM sucrose, 0.05 mM EDTA, and 10 mM Tris-HCl (pH 7.4) at 4°C. The homogenates were centrifuged at 600 g for 5 min and supernatants were centrifuged at 8800 g for 10 min. The pellet was washed twice with the same buffer. Protein concentration was determined using Coomassie Brilliant Blue [20].

### 2.6. Measurement of Mitochondrial Membrane Potential

The mitochondrial membrane potential (ΔΨ_m_) was evaluated according to Emaus et al. [21] by the uptake of the fluorescent dye rhodamine 123 (Rh123), which accumulates electrophoretically into energized mitochondria in response to the negative inside membrane potential. Isolated liver mitochondria were suspended in the assay buffer (0.5 mg protein/mL) containing 225 mM mannitol, 70 mM sucrose, 5 mM HEPES (*N*-2-hydroxyethylpiperazine-*N*-2-ethanesulfonic acid), pH 7.2. The mitochondrial membrane potential (ΔΨ_m_) was assessed spectrophotometrically (Hitachi 850) at 25°C with excitation at 505 nm and detection at 534 nm after addition of 0.3 *μ*M Rh123. The membrane potential was calculated by the relationship: *m* = −59 log [Rh123]_in_/[Rh123]_out_, assuming that the distribution of Rh123 between mitochondria and medium follows the Nernst equation [21].

### 2.7. Measurement of Mitochondrial Free Calcium

Intramitochondrial Ca^2+^ level was assayed by the Ca^2+^ indicator dye Fluo-3-acetoxymethyl (AM) ester. Briefly, an aliquot of the Fluo-3-AM stock solution in DMSO was mixed with an equal amount of Pluronic (Molecular Probes) and added to the assay buffer consisting HBSS, 20 mM HEPES, pH 7.4, and 2.5 mM probenecid (Molecular Probes) to give a final Fluo-3 concentration of 10 *μ*M. Pluronic helps to disperse the nonpolar AM ester in aqueous media, whereas probenecid (an inhibitor of organic anion transporters) reduces leakage of Fluo-3.

Liver mitochondria (0.5 mg protein/mL) of all experimental groups were incubated with the fluorescence Fluo-3-AM for 30 min at 37°C, and then washed twice to eliminate any free fluo-3-AM. The final mitochondria pellet was diluted in the suspension medium to obtain a protein concentration of 0.5 mg mL^−1^. Fluorescent intensity (*F*) of Fluo-3 loaded mitochondria was recorded on a Hitachi 850 fluorescence spectrometer at an excitation of 485 nm and an emission of 520 nm. The maximum fluorescsent intensity *F*_max_ was determined by adding 0.4% Triton X-100 and 1 mM CaCl_2_ and *F*_min_ was measured by adding 10 mM EGTA to the above system. The intra-mitochondrial Ca^2+^ content was calculated as follows: *K*_d_ (*F* − *F*_min_)/(*F*_max_ − *F*).

### 2.8. Determination of Mitochondrial Swelling

Mitochondrial swelling was assessed by measuring the absorbance at 540 nm. Liver mitochondria of all the experimental groups were prepared in the assay buffer (0.5 mg protein/mL) containing 125 mM sucrose, 50 mM KCl, 2 mM KH_2_PO_4_, 10 mM HEPES and 5 mM succinate. The extent of mitochondrial swelling was assayed by measuring the decrease in absorbance (*A*) at 1, 2, 3, 4, 5, 6, and 7 min after the addition of 100 *μ*M Ca^2+^ at 30°C and the swelling rate of mitochondrial swelling was calculated as follows: Δ*A*_drug_/Δ*A*_Control_ × 100%, Δ*A* = *A*_0min _ − *A* [22].

### 2.9. Evaluation of VDAC mRNA Level by Reverse Transcription-Polymerase Chain Reaction Assay

Total RNA was extracted from livers using RNAiso reagent. Reverse transcription (RT) was started with 5 *μ*g of total RNA at 37°C for 50 min in a 20 *μ*L reaction mixture containing 20 U RNase inhibitor, 0.25 mM each of dNTP, 0.5 *μ*g Oligo(dT)_15_ and 200 U M-MLV reverse transcriptase. The reaction was terminated by incubation at 70°C for 15 min. Polymerase Chain Reaction (PCR) amplification was performed for 28 cycles, by including 4 *μ*L cDNA by adding 5 mM MgCl_2_, 2.5 U Taq polymerase, 0.25 mM each dNTP and 5′- and 3′-sequence-specific oligonucleotide primers for VDAC and *β*-Actin in 10 × Taq polymerase reaction buffer, respectively. Each PCR cycle was set at 94°C, 30 s; 55°C, 50 s; 72°C, 1 min and finally at 72°C, 4 min. The internal standard was set with *β*-Actin. The amplified fragments were detected by agarose gel electrophoresis and visualized by ethidium bromide (EB) staining. The oligonucleotide primers used were: for VDAC, sense 5′-GGC TAC GGC TTT GGC TTA AT-3′ and anti-sense 5′-CCC TCT TGT ACC CTG TCT TGA-3′, yielding a deduced amplification product of 301 bps; whereas for *β*-Actin, sense 5′-AGT GTG ACG TTG ACA TCC GTA-3′ and anti-sense 5′-GCC AGA GCA GTA ATC TCC TTC T-3′′ yielding a deduced amplification product of 112 bps.

### 2.10. Western Blot Analysis for VDAC

Liver samples were homogenized in ice-cold lysis buffer. Homogenates were centrifuged at 12 000  g for 10 min and the supernatants were collected, and the protein concentration was determined using Coomassie Brilliant Blue. The samples (50 *μ*g per lane) were dissolved in the sample buffer and separated by 12% SDS-polyacrylamide gel electrophoresis (PAGE) gels and electrophoretically transferred onto a polyvinylidene-difluoride (PVDF) membrane (Bio-Rad). The membrane was incubated with VDAC primary antibody (1 : 4000) and *β*-Actin antibody (1 : 80 000). The membrane was then exposed to the enhanced chemiluminescence (ECL) solution.

### 2.11. Statistical Analysis

Differences among all groups were analyzed by one-way analysis of variance (ANOVA), followed by SNK-*q*-test; a *P* value < .05 was accepted as statistically significant. All experiments were repeated at least three times.

## 3. Results

### 3.1. sAST and sALT Activities

Serum enzyme activity of mice in the prescriptions of MCE is shown in Table 1. Serum ALT and AST activities increased remarkably (7.8-fold and 2.0-fold, resp.) after the CCl_4_ injection. However, treatment with various concentrations of MCE (50, 150, and 450 mg kg^−1^) blocked the above changes significantly in a dose-dependent manner, and the elevations in sALT and sAST activities were almost completely inhibited by MCE_H_.

### 3.2. Histological Observation

**Figure 2:**
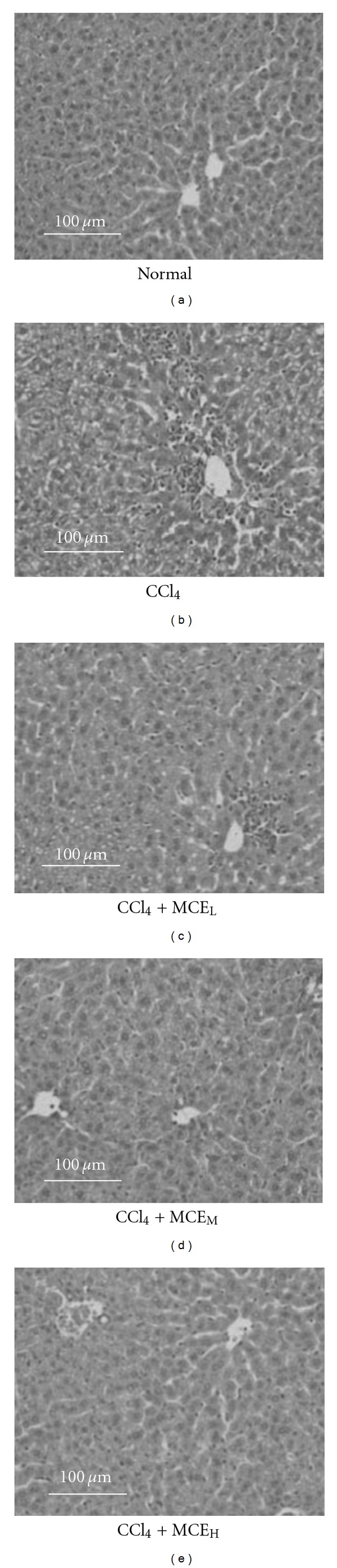
Photomicrographs of liver sections taken from mice treated with CCl_4_ with or without pretreatment with MCE. (a) Normal; (b) CCl_4_; (c) CCl_4_ + MCE_L_; (d) CCl_4_ + MCE_M_; and (e) CCl_4_ + MCE_H_.

The histological changes associated with the hepatoprotective activity in three prescriptions of MCE basically supported the estimation of the serum enzyme activities. The livers of the CCl_4_-intoxicated mice showed gross necrosis, broad infiltration of the lymphocytes and Kupffer cells around the central vein and loss of cellular boundary (Figure 2). The histological pattern of the livers of the mice treated with MCE only showed mild degrees of necrosis and lymphocyte infiltration. MCE_H_ pretreatment significantly prevented CCl_4_-induced inflammatory infiltration to the extent almost the same as that in the normal group (Figure 2). 

### 3.3. Protection on the Ultrastructure of Hepatocytes

**Figure 3:**
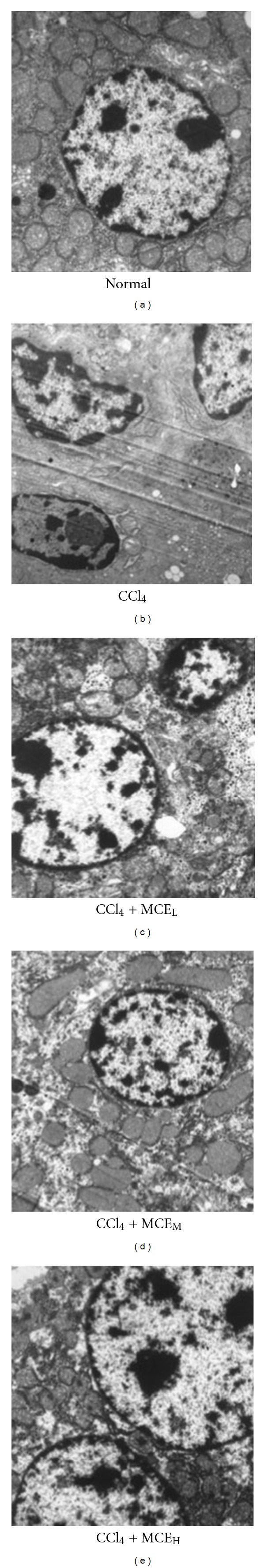
Protective effect of MCE on the ultrastructure of hepatocytes induced by CCl_4_. Mice were divided into five groups: (a) Normal, (b) CCl_4_, (c) CCl_4_ + MCE_L_, (d) CCl_4_ + MCE_M_ and (e) CCl_4_ + MCE_H_. The CCl_4_ and different MCE groups were administered saline or various concentrations of MCE orally for 5 days before the intra-peritoneal injection of 0.30% CCl_4_. Specimens were taken 24 h later and regularly prepared for examination under an electron microscope (×5000).

Obvious ultrastructural changes in mouse hepatocytes showing mitochondrial disintegration, lipid deposition and nuclear condensation as compared with the control (Figure 3) were induced by CCl_4_. However, the structure in mice treated with MCE_L_ was improved to some extent, and in the MCE_H_ group the hepatocytes were similar to normal cells (Figure 3).

### 3.4. Mitochondrial Membrane Potential

**Figure 4:**
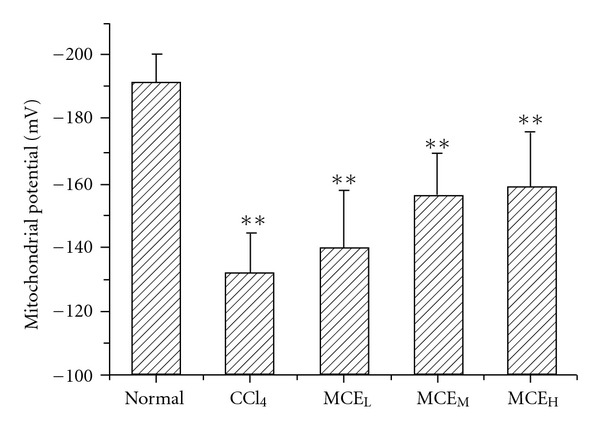
Prevention of MCE on mitochondrial membrane potential dissipation induced by CCl_4_. Mice were treated with MCE for 5 days before pretreatment with CCl_4_. Liver mitochondria were isolated and the mitochondrial membrane potential was determined using Rh123. Each value represents mean ± SD (*n* = 8). *P < .05, **P < .01, versus Normal; ^+^ *P* < .05 versus CCl_4_ group.

Under the present experimental conditions, the mitochondrial membrane potential of the normal mice was −191.94 ± 8.84 mV. This value dropped to −132.06 ± 12.26 mV (*P* < .01) after the mice had been intra-peritoneally injected with CCl_4_ (Figure 4). MCE attenuated CCl_4_-induced mitochondrial membrane potential dissipation in a dose-dependent manner. At a dose of 150 or 450 mg kg^−1^ of MCE, the mitochondrial membrane potentials were restored (*P* < .05).

### 3.5. Intra-Mitochondrial Free Calcium

**Figure 5:**
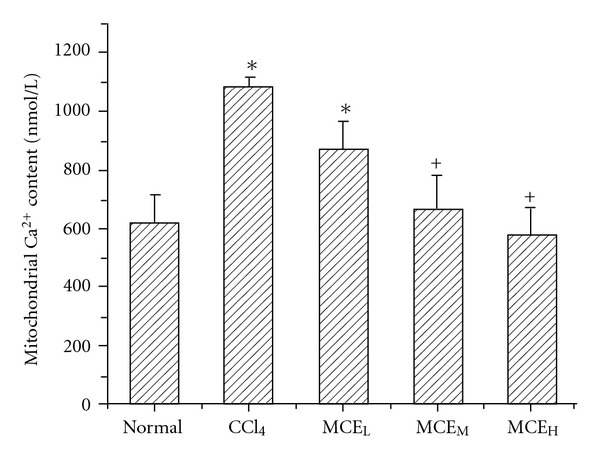
Effect of MCE on liver mitochondrial calcium content in mice treated with CCl_4_. Mice were treated with MCE for 5 days before pretreatment with CCl_4_. Liver mitochondria were then isolated and mitochondrial free calcium content was determined using Fluo-3. MCE showed a dose-dependent suppression of the CCl_4_-induced intra-mitochondrial Ca^2+^ overload. Values represent mean ± SD (*n* = 6). *P < .01 versus the normal group; ^+^ *P* < .01 versus the CCl_4_ group.

Measurement of calcium content using the fluorescent probe Flou-3 showed that intra-mitochondrial free calcium concentration in the CCl_4_-intoxicated mice was higher (1.8-fold, *P* < .05) than that in the normal mice. However, the elevation in calcium level induced by CCl_4_ was significantly inhibited by pretreatment with MCE_L_, MCE_M_ or MCE_H_, and the MCE_H_ group's mitochondrial calcium content was maintained at the normal level (Figure 5).

### 3.6. Ca-Induced Mitochondrial Swelling

**Figure 6:**
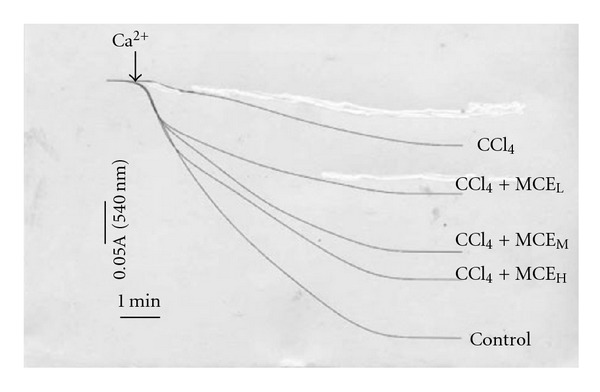
Effect of MCE on Ca^2+^-induced mitochondrial swelling. CCl_4_ can decrease the sensitivity in Ca^2+^-induced mitochondrial swelling. MCE dose-dependently recuperated this sensitivity. The curves represent typical recordings from experiments of at least three different mitochondrial preparations.

The swelling of liver mitochondria, which could be attenuated by treatment with CCl_4_, was induced by 100 *μ*M Ca^2+^. The swelling rates of MCE_L_, MCE_M_ and MCE_H_ at 7 min were 43.4%, 66.1%, and 77%, respectively, which were more sensitive than that of CCl_4_ (24.3%) (Figure 6).

### 3.7. Prevention against Reduction of Liver VDAC Expression

**Figure 7:**
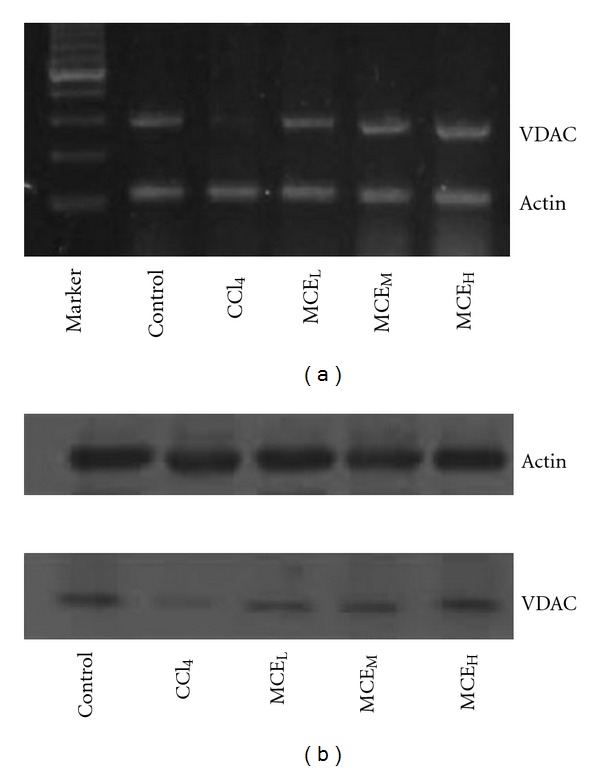
Effect of MCE on mitochondrial VDAC expression in CCl_4_-insulted mouse livers. Livers from various groups were taken 24 h following 0.30% CCl_4_. (a) Inhibitory effect of MCE on the decrease in VDAC mRNA level induced by CCl_4_ analyzed by RT-PCR. (b) Inhibitory effect of MCE on the decrease in VDAC protein level induced by CCl_4_ analyzed by western blot.

The effect of MCE on VDAC transcription was examined by RT-PCR. As shown in Figure 7(a), the expression of VDAC mRNA was detected in the normal group, but a lower level of VDAC mRNA was detected when the mice were stimulated with 0.3% CCl_4_. Furthermore, MCE_L_, MCE_M_, and MCE_H_ significantly blocked the CCl_4_-stimulated VDAC mRNA reduction.

MCE up-regulation on VDAC protein expression was further corroborated by western blot (Figure 7(b)). Normal mouse livers showed a strong signal for VDAC, and mice receiving CCl_4_ alone showed a significant decrease. In contrast, compared with mice treated with CCl_4_ alone, in mice pre-administrated with MCE, a stronger VDAC protein band occurred 24 h following CCl_4_ treatment.

## 4. Discussion

The liver has versatile functions and plays important roles in metabolism, such as biosynthesis of plasma proteins, gluconeogenesis and detoxification. Although the liver has strong regeneration ability, when cellular loss exceeds a certain threshold, the insufficient functions cause hepatic failure, leading to liver disease. Overdose of drug or ischemia/reperfusion induces necrotic and apoptotic cell death of hepatocytes and non-parenchymal liver cells. One promising approach to prevent liver injury or to treat patient with liver disease is to confer resistance against cell death on hepatocytes and non-parenchymal cells.

The results of the present study show that 50, 150, or 450 mg kg^−1^ MCE significantly protects mice against CCl_4_-induced hepatotoxicity as demonstrated by its inhibition of the elevation of sAST and sALT. CCl_4_-induced acute liver injury may be initiated by the •CCl_3_ radical, which is formed by a metabolic enzyme (cytochrome P450) and could induce peroxidation of the unsaturated fatty acids of cell membrane, and lead to membrane injury and leakage of enzymes such as AST and ALT [23]. In fact, sAST and sALT are the most sensitive indicators of liver injury, with the extent of hepatic damage assessed by the serum level of enzymes released from cytoplasm and especially mitochondria. It has been demonstrated that ALT enzyme is one of the indices of the degree of cell membrane damage, whereas AST is one of the indices of mitochondrial damage since mitochondria contain 80% of the enzyme [24]. Based on our results, we speculated that MCE has a protective effect on both hepatocytes and their mitochondria. This was confirmed by ultrastructure examination. Protection of liver mitochondria against hepatocytes injury induced by CCl_4_ was demonstrated for MCE (150 and 450 mg kg^−1^).

It is now generally accepted that maintenance of mitochondrial membrane potential is necessary for mitochondria to carry out their functions. In the present work, the effect of MCE on liver mitochondrial membrane potential in CCl_4_-intoxicated mice was assessed. Treatment of mice with CCl_4_ damaged the liver mitochondria as characterized by the dissipation of mitochondrial membrane potential which is in agreement with a previous report [25]. MCE could prevent the collapse of mitochondrial membrane potential, which confirmed the suggestion of MCE's protective effect against mitochondria deficiency.

It is very crucial for the cell to maintain cytosolic Ca^2+^ at very low levels (0.1–0.2 *μ*M) and CCl_4_ can result in hepatocellular Ca^2+^ overload [26], which can activate the mitochondrial Ca^2+^ uniporter in the mitochondrial inner membrane, induce a mitochondrial Ca^2+^ influx and finally damage mitochondria [27]. In the examination, we evaluated the effect of MCE on the intra-mitochondrial Ca^2+^ content in the CCl_4_-intoxicated mice. MCE showed a dose-dependent suppression of the CCl_4_-induced intra-mitochondrial Ca^2+^ overload. This suggested an important relation between the preserving effect of MCE on mitochondrial calcium homeostasis and its protection of liver mitochondria.

Mitochondria isolated from mice livers were used to assess the effect of MCE on Ca^2+^-induced liver mitochondria permeability transition (MPT) to search for the possible mechanisms of MCE protection of liver mitochondria. Ca^2+^-induced liver MPT is a useful model for evaluating the effects of drugs or other substances on mitochondrial PTP [28, 29]. MPT could be induced by 100 *μ*mol/L Ca^2+^ in liver mitochondria freshly isolated from mice. It was also found that the decreased sensitivity in Ca^2+^-induced mitochondrial swelling, induced by CCl_4_, could be prevented by MCE. This indicates a protective role of MCE on normal MPT of mitochondria.

Furthermore, there was accumulating evidence that there were changes in the levels of expression of the mitochondrial VDAC, one of the most important proteins on the outer membrane regarding the process of apoptosis [30–32]. VDAC levels decreased significantly after CCl_4_ administration and pretreatment of MCE could dose-dependently inhibit the reduction of both transcriptional and translational levels of VDAC in acute liver injury process, suggesting that the protective effect of MCE on liver mitochondrial in mice might be related to an up-regulation of the expression of mitochondrial VDAC which could be decreased by CCl_4_.

**Figure 8:**
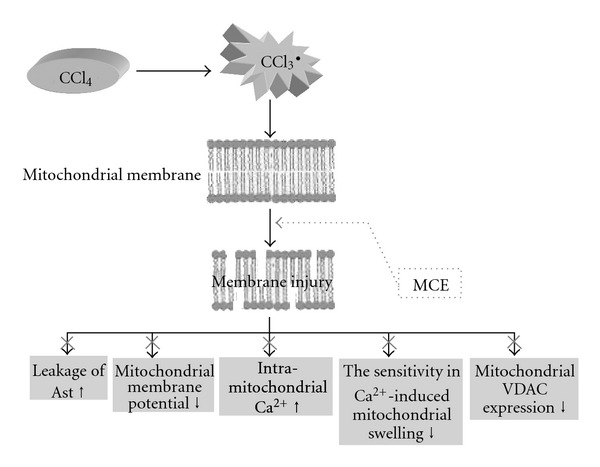
CCl_4_ could induce peroxidation of the unsaturated fatty acids of cell membrane, and lead to membrane injury, enhance the leakage of AST, the dissipation of mitochondrial membrane potential, hepatocellular Ca^2+^ overload and decrease the sensitivity in Ca^2+^-induced mitochondrial swelling and other the mRNA and the protein of VDAC's expressions. Using MCE could prevent all these changes, that is to say, MCE has hepatoprotective activity and the mechanisms underlying its protective effects may be related to the mitochondrial protection.

In conclusion, the results of the present study suggested that MCE has hepatoprotective activity and the mechanisms underlying its protective effects may be related to the mitochondrial protection and especially the regulation of VDAC expression (Figure 8). The active components of MCE await further study. 

## Figures and Tables

**Table 1:** Effect of MCE on CCl_4_-induced acute liver damage in mice.

Groups	Animals (*n*)	sALT (enzyme unit/100 mL serum)	sAST (enzyme unit/100 mL serum)
Normal	8	36.4 ± 8.4	82.1 ± 23.1
CCl_4_	8	283.3 ± 128.1**	164.9 ± 41.3**
CCl_4_ + MCE_L_	8	198.7 ± 101.9^∗†^	128.8 ± 40.1^†^
CCl_4_ + MCE_M_	8	148.2 ± 78.4^∗†^	106.9 ± 47.1^††^
CCl_4_ + MCE_H_	8	33.6 ± 7.6^††^	75.4 ± 25.5^††^
CCl_4_ + DDB	8	33.5 ± 9.1^††^	96.90 ± 32.4^††^

*P < .05 versus Normal, **P < .01 versus Normal, ^†^ *P* < .05 versus CCl_4_ group, ^††^ *P* < .01 versus CCl_4_ group
